# A gene pathogenicity tool “GenePy” identifies missed biallelic diagnoses in the 100,000 Genomes Project

**DOI:** 10.1016/j.gim.2024.101073

**Published:** 2024-01-18

**Authors:** Eleanor G. Seaby, Gary Leggatt, Guo Cheng, N. Simon Thomas, James J. Ashton, Imogen Stafford, Diana Baralle, Heidi L. Rehm, Anne O’Donnell-Luria, Sarah Ennis

**Affiliations:** 1 Human Development and Health, Faculty of Medicine, University Hospital Southampton, Southampton, Hampshire, United Kingdom; 2 Program in Medical and Population Genetics, Broad Institute of MIT and Harvard, Cambridge, MA; 3 Division of Genetics and Genomics, Boston Children’s Hospital, Boston, MA; 4 Paediatric Infectious Diseases, Imperial College London, London, United Kingdom; 5 Wessex Regional Genomics Laboratory, Salisbury NHS Foundation Trust, Salisbury, United Kingdom; 6 University of Surrey, Guildford, Surrey, United Kingdom; 7 Center for Genomic Medicine, Massachusetts General Hospital, Boston, MA

**Keywords:** Diagnostic uplift, Next-generation sequencing, Novel methods, Rare disease, Recessive disease

## Abstract

**Purpose:**

The 100,000 Genomes Project diagnosed a quarter of affected participants, but 26% of diagnoses were not on the applied gene panel(s); with many being de novo variants. Assessing biallelic variants without a gene panel is more challenging.

**Methods:**

We sought to identify missed biallelic diagnoses using GenePy, which incorporates allele frequency, zygosity, and a user-defined deleterious metric, generating an aggregate GenePy score per gene, per participant. We calculated GenePy scores for 2862 recessive disease genes in 78,216 100,000 Genomes Project participants. For each gene, we ranked participant GenePy scores and scrutinized affected participants without a diagnosis, whose scores ranked among the top 5 for each gene. In cases which participant phenotypes overlapped with the disease gene of interest, we extracted rare variants and applied phase, ClinVar, and ACMG classification.

**Results:**

3184 affected individuals without a molecular diagnosis had a top-5-ranked GenePy score and 682 of 3184 (21%) had phenotypes overlapping with a top-ranking gene. In 122 of 669 (18%) phenotype-matched cases (excluding 13 withdrawn participants), we identified a putative missed diagnosis (2.2% of all undiagnosed participants). A further 334 of 669 (50%) cases have a possible missed diagnosis but require functional validation.

**Conclusion:**

Applying GenePy at scale has identified 456 potential diagnoses, demonstrating the value of novel diagnostic strategies.

## Introduction

The 100,000 Genomes Project (100KGP) was a UK-government-funded research project led by Genomics England (GEL) to sequence 100,000 genomes for families predominantly presenting with rare disease.^1^ The project utilized a phenotype to genotype approach, whereby genome sequencing data were filtered using a pre-selected PanelApp^2^ gene panel or panels chosen by GEL based on the Human Phenotype Ontology (HPO)^3^ terms recorded at recruitment.^1,4^ The project was completed in 2020 and yielded an overall diagnostic rate of ~25% across all rare-disease categories.^1,5^ However, as ever-increasing numbers of researchers gained access to anonymized genome sequencing data from the 100KGP, additional diagnoses were made using methods that extended variant analysis beyond gene panels across more coding and non-coding regions, which have subsequently been returned to participants.^4^ As of 2022, 26% of all diagnoses returned by the 100KGP were from diagnoses not on the pre-selected gene panel applied, with many being pathogenic de novo coding variants.^4,5^ However, assessing other variants such as biallelic variants is more burdensome, particularly without the use of gene panels because of the sheer number of variants that require scrutiny. This is because many are inherited from unaffected relatives and are carried at non-trivial allele frequencies in population databases. Furthermore, biallelic variation is often hard to interpret, especially for compound heterozygotes, in which one variant may be pathogenic, and another may be a copy-number variant, non-coding variant, or other variant of uncertain significance (VUS). This is where gene panels show their greatest utility because they can help narrow down variants to clinically relevant genes.^2^ However, this approach must be balanced against the potential of missing diagnoses outside of the original gene panel applied.

We sought to identify potential missed biallelic diagnoses in recessive disease genes independently of the gene panel applied using a genome pathogenicity metric called GenePy, pronounced “Jenni-pea (dƷ′εnɪp,i:).” GenePy (https://github.com/UoS-HGIG/GenePy-1.3) is a gene pathogenicity prioritization tool developed at the University of Southampton that transforms the interpretation of next generation sequencing data from the variant level to the gene or pathway level.^6^ GenePy incorporates allele frequency, individual zygosity (in which a heterozygote scores 1 point and a homozygote scores 2 points), and a user-defined deleterious metric (such as the Combined Annotation Dependent Depletion [CADD] score^7^) into a single variant score.

GenePy is defined as follows:

$$S_{gh}=-\sum_{i=1k}D_{i}\log_{10}\left(f_{i1}\cdot f_{i2}\right)$$

(in which h = individual; g = gene; k = variants; i = locus; $D_{i}$ = allele deleteriousness; $f_{i}$ = allele frequency; $f_{i1}$ = allele 1; $f_{i2}$ = allele 2).

**Figure 1:**
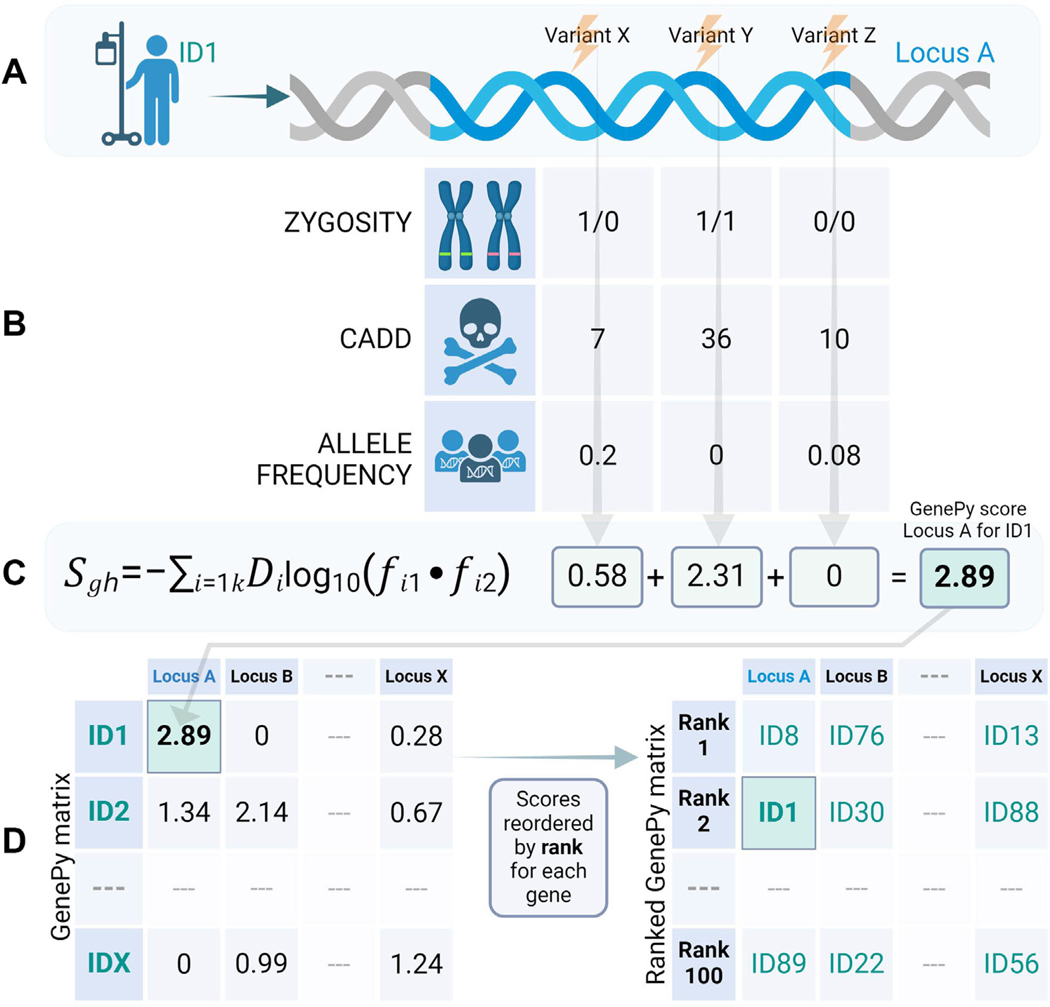
Overview of GenePy pathogenicity software and output. A. Patient’s DNA undergoes sequencing and subsequent processing to produce a file listing all variants identified in their data. B. Each variant is individually annotated with biological information reflecting: zygosity, ie, the allele inherited from each parent, deleteriousness (D, we commonly use a metric called CADD, but this can be user specified), and frequency of the observed alleles (*f*) for which we refer to gnomAD—one of the largest population database resources reporting the observed occurrence of alleles across very large population datasets. C. These data are input into the GenePy algorithm for each variant and then summed across all variants observed within that gene for that individual. This step is run in parallel for all genes across all patients within the cohort. D. The output is a matrix of all individuals by all genes. For certain applications, this matrix can be transposed such that for each gene, individuals are ordered by highest pathogenic variant loading.

GenePy then aggregates variant scores across genes in an additive manner, generating a single score, per gene, per individual that is represented in a GenePy matrix table (Figure 1). However, for large genes and intronic regions there is a potential to accumulate noise from low scoring variants. To mitigate this, GenePy can be customized to filter variants with high in silico scores only, eg, CADD score above a particular threshold. Additionally, GenePy can be applied across any defined interval and variant scores do not have to be summed across genes, eg, one may choose to sum variants across a particular biological pathway or genomic region.

Upon generation of a GenePy matrix, GenePy scores can be compared across individuals in a cohort; GenePy scores are intuitive in that higher GenePy scores correlate with higher pathogenic variant burden such that individuals can be ranked for their score for any given gene, relative to all individuals with comparable input genomic data. GenePy scores are not easily compared between genes, without normalization and adjustment for gene length. Even then, genes with alternative tolerance to dysfunctional variation are likely to exhibit very different GenePy score profiles. Instead, GenePy demonstrates the greatest utility when individual gene scores are compared across large numbers of individuals. Because GenePy is an additive score, individuals in large cohorts with the highest ranked GenePy scores will be enriched for biallelic disease. Given the potential for missed biallelic diagnoses in the 100KGP, we applied GenePy at scale in a panel-agnostic way to uplift diagnostic rates.

## Materials and Methods

### Access to 100KGP data

Participants were recruited to the 100KGP with written consent. The full protocol is available here: https://doi.org/10.6084/m9.figshare.4530893.v7. Deidentified data from the project are held in the secure Genomics England Research Environment (RE).

We obtained access to 100KGP data after governance training and through membership of the “*Quantitative Methods, Machine Learning, and Functional Genomics*” Genomics England Clinical Interpretation Partnership. We had an approved Genomics England Project (RR359).

In 2022, we accessed 78,216 genomes from affected and unaffected participants recruited to the 100KGP. We extracted participants’ affection status (ie, whether they were coded as affected with disease or not) and any HPO terms associated with participants’ records. Using the package LabKey in R, we queried the “GMC Exit Questionnaire” SQL table and extracted any diagnostic (likely pathogenic/pathogenic) variants returned to participants by the project.

### Curating a list of recessive disease genes

To target our method toward potential missed biallelic diagnoses, we curated a list of 2862 recessive disease genes using the Online Inheritance in Man (OMIM)^8^ database (downloaded in May 2022) and cross checked these findings with the Gene Curation Coalition (GenCC) database, whereby discrepancies in inheritance were examined more carefully.^9^ We then generated a bed file of gene coordinates for GRCh38 using the UCSC Genome Browser. The full gene list is available in Supplementary File 1.

### Application of GenePy

Within the Genomics England RE we applied GenePy v.1.3 (https://github.com/UoS-HGIG/GenePy-1.3) software to 78,216 participants in the 100KGP using CADD^7^ v1.6 as our deleterious metric and the gnomAD v.2.1.1 and v.3^10^ databases as our reference for allele frequency. We selected variants with a minimum depth of 10, minimum genotype quality (GQ) of 20, and mean GQ > 35 using vcftools. We applied a call-rate filter, whereby each variant had to be genotyped in at least 70% of the cohort. For downstream analysis, we only modeled and scored participant variants annotated as coding +/− 8 base pairs (on any transcript) and with a CADD score ≥15. We specified CADD as our input metric because it scores the greatest variety and number of variant types. We generated GenePy scores for 2862 recessive disease genes to create a matrix comprising GenePy scores for 2862 genes across 78,216 individuals. Of note, in addition to “affected” participants, this cohort included many “control”-type individuals that represented unaffected parents of affected children and germline genomes of cancer patients.

**Figure 2:**
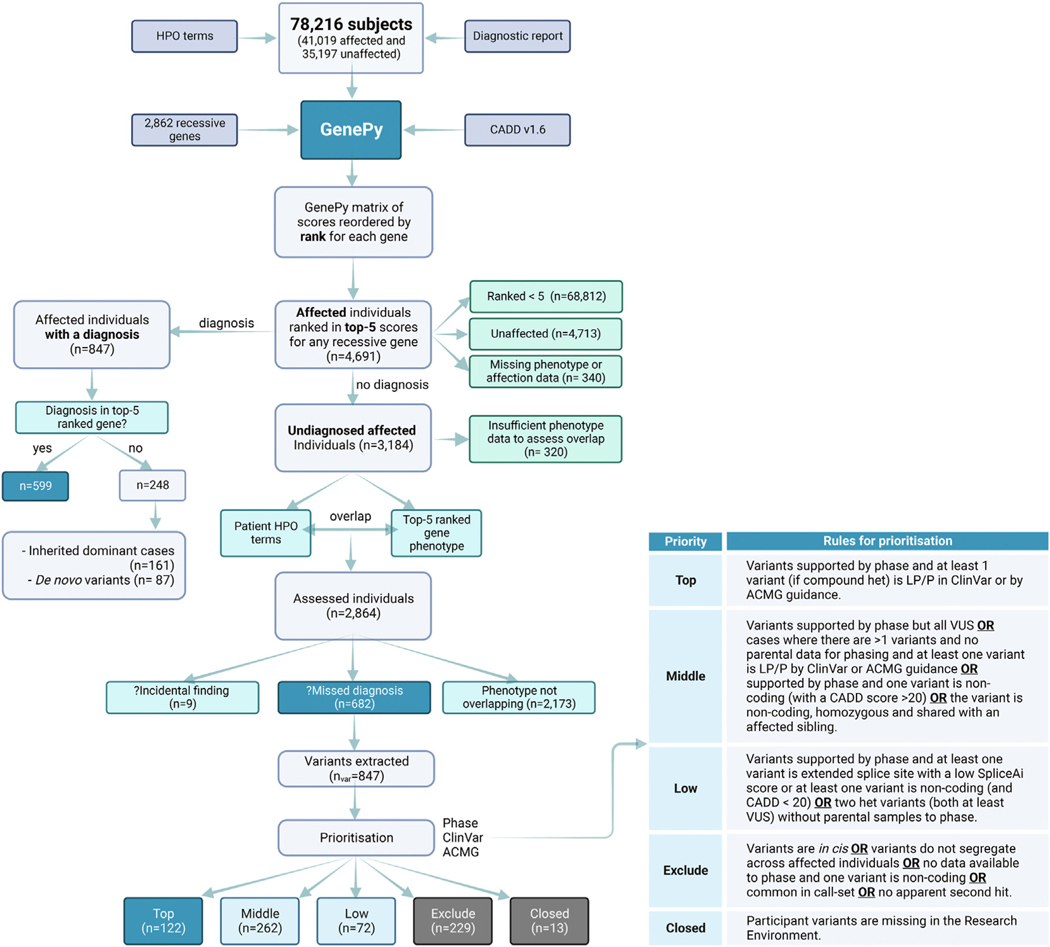
Workflow of GenePy applied to 78,216 participants in the 100,000 Genomes Project. GenePy scores were created for 2862 autosomal recessive genes in 78,216 participants, using CADD v.1.6 and gnomAD v.2.1.1. Participants scores were ranked across the cohort per gene, whereby those who ranked in the top 5 GenePy score for each gene were retained for downstream analysis. Unaffected individuals were removed. HPO terms from unaffected individuals without a diagnosis returned by the 100,000 Genomes Project were compared with the clinical features described for the autosomal recessive gene that the participant scored in the top 5 for. If the participant’s HPO terms overlapped with the gene that the person ranked in the top 5 for, we extracted the individual participant variants and assessed phase and ClinVar status and applied ACMG guidelines. We then prioritized the findings according to the prioritization rules, with “Top” priority being putative missed diagnoses, “Middle” and “Low” priority being of interest but lacking sufficient evidence, “Exclude” being not diagnostic and “Closed” being when the participants had been withdrawn from the Project.

For each of the 2862 recessive genes, we ranked every Genomics England participant’s GenePy score relative to one another, eg, the person with the highest GenePy score for *CFTR* would be ranked 1, and the person with the lowest GenePy score in *CFTR* would be ranked 78,216. After ranking, we arbitrarily assessed only individuals who ranked among the top-5 GenePy score for each gene. If 2 individuals had identical scores, we retained all participants with a rank of 5 or less. We then removed any individuals who were coded as unaffected, and affected individuals with insufficient phenotypic data in the form of HPO terms recorded. We next separated affected cases into those with a confirmed diagnosis returned by the 100KGP and those with a negative result. If the participant had a diagnosis returned, we assessed whether the established diagnostic variant was in a gene with a top-5 ranked GenePy score (Figure 2).

For affected participants with a negative genome result, we extracted HPO terms from R LabKey and compared these HPO terms with the clinical features associated with the disease gene for which they scored in the top 5 rank. For example, if the participant had the HPO terms “pancreatic insufficiency,” “failure to thrive,” and “recurrent chest infections” and they ranked 3rd for *CFTR*, we would compare their HPO terms with the clinical features of cystic fibrosis. This process was completed manually by a clinician who used clinical acumen, phenotypic descriptions and HPO terms listed in OMIM, and the clinical literature to help assess phenotype overlap. If the participant’s HPO terms were consistent with those for a gene that the same participant was ranked in the top 5 GenePy scores for (eg, the participant had pancreatic insufficiency and recurrent chest infections and was ranked 3rd in *CFTR)*, this was considered a potential missed diagnosis. If the disease-gene phenotype was unrelated to the participant’s clinical phenotype but represented a gene in the American College of Human Genetics and Genomics (ACMG) 78^11^ list or may represent an adult-onset disease, this was considered a potential incidental finding. For these, we contacted the recruiting clinician to discuss the findings. If there was no correlation between the participant’s HPO terms and the clinical phenotype for the implicated disease gene, this was considered to be lacking phenotypic overlap and excluded from further consideration.

### Assessing potential missed diagnoses

When the participant’s phenotype was overlapping with the disease gene for which the participant ranked in the top 5, we extracted all variants from the participant’s variant call file with a CADD score ≥15. These variants were then prioritized by likelihood of being a missed biallelic diagnosis, taking into consideration variant phase where possible, ClinVar^12^ status, and variant curation by ACMG/AMP^13^ guidelines (Figure 2). Variants that were prioritized as “Top” priority were considered putative missed diagnoses and mostly represented homozygous likely pathogenic/pathogenic variants or likely pathogenic/pathogenic com- pound heterozygous variants.

## Results

We applied GenePy to 2862 recessive disease genes in 78,216 participants recruited to the 100KGP (Figure 2). For each gene we selected the top 5 ranked participants by GenePy score, which yielded a total of 9404 unique participants, with some participants ranking top 5 for more than one recessive gene. Of the top ranked participants, 4713 of 9404 (50.1%) were unaffected and 4691 of 9404 (49.9%) were affected. Unaffected participants (rare disease or cancer germline) represented 45% of the entire cohort. Of the 4691 affected participants with a top-5 ranked GenePy score, 847 of 4691 (18.1%) already had a diagnosis returned by the 100KGP up to 2022. Of these, 599 of 847 (70.7%) had diagnoses in 1 of the top 5 ranked genes. Twenty-nine percent (248/847) of individuals had a diagnosis returned by GEL in an alternative gene and all of these diagnoses were returned as complete diagnoses (ie, they explained the entire phenotype). Of these, 87 individuals had a de novo pathogenic variant and 161 had a pathogenic variant in a dominant gene (either inherited from an affected individual or the participant was a from a singleton family).

In total, there were 3184 affected individuals who had a “no diagnosis” genome report returned by the 100KGP who were ranked in the top 5 GenePy scores for the 2862 computed recessive disease genes. For these cases, we compared the participant’s reported HPO terms with the clinical phenotype of the GenePy disease gene implicated in the participant. For 340 participants, there were missing phenotype data—typically this was an affected relative with no HPO terms. For 320 participants, there was insufficient HPO terms recorded to assess for phenotypic overlap between the participant’s clinical phenotype and that of the implicated disease gene. These were either due to a very limited number of non-specific HPO terms or only 1 HPO term recorded. Therefore, these individuals were removed from downstream analysis. There were 2864 individuals who had sufficient HPO terms to assess phenotype overlap and for 682 of 2864 (23.8%) of these cases, the participant’s HPO terms overlapped with the clinical presentation associated with the top 5 ranked GenePy disease gene. For 2173 of 2864 (75.9%) of cases, the phenotypes were non-overlapping and for 9 of 2864 (0.3%) of cases the phenotypes were not overlapping, but the implicated gene was 1 of the ACMG 78 incidental finding genes.

For the 682 participants with a potential missed diagnosis, we extracted variants in their top 5-ranked gene with a CADD score ≥15 directly from their variant call file. In total we extracted 847 unique variants. Following prioritization (Figure 2), we identified 122 top priority, putative missed diagnoses supported by phase, ClinVar^12^ classifications and ACMG/AMP guidelines (Supplemental Results).^13^ 262 individuals were assigned “Middle” priority demonstrating supportive evidence for a potential missed diagnosis, whereby for many there was lack of phased data limiting diagnostic potential. Seventy-two individuals had some, but weak, evidence for a potential missed diagnosis, for example, because of 1 variant being non-coding on the matched annotation from NCBI and EMBL-EBI (MANE)^14^ transcript and were assigned “Low” priority. Two hundred twenty-nine cases were ruled as non-diagnostic, typically because of the variants being in *cis*, being non-coding on the MANE transcript, not segregating with affected and related individuals, and being common in the 100,000 Genomes call-set (Table 1). There were 3 cases in which 1 variant was a predicted loss-of-function (pLoF), and the second variant was non-coding on the MANE transcript (Supplemental Results). Alternative transcripts were considered for these 3 cases; however, the coding transcripts had poor overall expression in gnomAD. In 13 cases, no variants were extracted because the individual had withdrawn from the 100KGP.

## Discussion

We applied a gene pathogenicity score, GenePy, to a cohort of 78,216 individuals recruited to the 100KGP. Utilizing ranked individuals’ GenePy scores for 2862 recessive disease genes, we identified outliers with the highest GenePy scores per gene. We selected individuals who ranked in the top 5 scores for each gene, with an expectation that these individuals may harbor missed biallelic diagnoses.

Eight-hundred and forty-seven individuals with a top 5 ranked GenePy score had a diagnosis returned by the 100KGP. Seventy-one percent (599/847) of these individuals had a diagnosis in a top 5 ranked gene, demonstrating how GenePy was able to rapidly recover 71% of diagnoses, showing potential diagnostic utility for both known and novel disease genes. The remaining 248 cases had diagnoses in dominant genes, with 81 diagnoses being de novo and 161 being inherited from an affected individual or the individual represented a singleton.

In total we identified 2864 undiagnosed individuals with top 5 ranked GenePy scores, of which 682 of 2864 (24%) had phenotypes overlapping with the clinical features of their top ranked recessive disease gene. Following prioritization and removing 13 cases in which participants had withdrawn from the 100KGP, 122 of 669 (18%) of the phenotype-matched cases had a putative missed diagnosis supported by phase, ClinVar classifications and ACMG/AMP guidelines. All these findings have since been returned to GEL through their Diagnostic Discovery Pathway. For 334 of 669 (50%) of individuals, we identified variants of interest in a disease gene consistent with the participant’s phenotype with some supportive evidence for pathogenicity, but often phase could not be determined because of missing parental data. Additionally, for many of these cases, the variants contributing to the high GenePy scores were classified as VUS and therefore require additional functional work-up. These variants are being reviewed by a clinical scientist in an NHS accredited diagnostic laboratory. Although follow-up of these variants is outside the scope of this research project, many of these variants, even those prioritized in the low category, may represent pathogenic variants. For example, non-coding variants were assigned to a lower priority grouping, despite them having a CADD score ≥15. It is hoped that many of these variants may be functionally investigated in the future as high-throughput methods to model VUS advance.

Application of GenePy has identified putative missed diagnoses, which raises the question as to why these were not detected and returned by the 100KGP. For the 100KGP, referring clinicians recorded HPO terms, but the number recorded was very variable; some patients only had 1 or 2 non-specific terms recorded. The in silico gene panel selection was made by GEL based on the HPO terms provided. This was a major limitation of the 100KGP; indeed, 26% of all diagnoses made from the project were not on the original panel applied.^5^ This showcases the limitations of panel-based strategies and highlights the need for panel-agnostic methods such as GenePy to recover missed diagnoses.

In total, GenePy has identified potential missed diagnoses in 456 of 2864 (16%) of undiagnosed individuals who had a top-5 ranked GenePy score in a recessive disease gene. Forty-eight variants were previously identified as VUS by GEL (Supplemental Results). On average this resulted in the curation of 1.2 additional variants per participant. Therefore, the application of GenePy successfully uplifted diagnosis rates without adding large variant numbers requiring time-consuming manual curation for diagnostic laboratories to assess and classify.

GenePy^6^ is an open-source transferrable piece of software that can be successfully applied at scale. GenePy matrices can be used as reference datasets for other cohorts applying the same GenePy methods, ie, when applying the same deleterious metric, population reference database, and quality control thresholds. For example, GenePy may be applied to a cohort of 10 samples, whereby these 10 individuals’ GenePy scores could be ranked against a larger GenePy matrix comprising 100,000 individuals. However, GenePy matrices for genome sequencing data should only be compared with other genome sequencing datasets, unless restricted to the same target regions of exome data.

### Limitations and opportunities

The application of GenePy to the 100KGP is not without its limitations. For one, we used an entirely arbitrary cutoff of 5 when we ranked individuals. It is entirely possible that a more permissive value may capture a wider range of diagnoses; however, this must be balanced with the additional number of variants, per individual, which would require further scrutiny by clinical laboratories.

We assessed for phenotype overlap between the participants’ HPO terms and the clinical features described for the disease gene in which the participants ranked in the top 5 GenePy scores. For 320 cases, the HPO terms were so limited (sometimes only 1 HPO term was recorded) that it was not possible to assess overlap. This represents a real-world limitation of sequencing studies in which there is often variability in how submitters record phenotype data and highlights the importance of accurate phenotyping. This phenotype comparison step was performed manually on 2864 cases. This large number of cases required 4 weeks of manual curation. Application of automated methods to compare participant HPO terms with disease-gene phenotypes may, in the future, has the potential to increase efficiency for GenePy applied at scale. However, it is unlikely that clinical or diagnostic laboratories applying GenePy would be reviewing thousands of individuals at once, but rather on a case-by-case basis. Additionally, automated methods lack the clinical knowledge and experience of a clinician or clinical scientist that may be better able to intelligently compare groups of similar phenotypes.

In our application of GenePy we used CADD v.1.6 to capture and model the greatest breadth of variation in an unbiased way, but it may be that incorporation of other metrics for different variant types (eg, REVEL^15^ for missense) may prove more sophisticated in an improved model. However, this is likely to require machine learning to apportion in silico weightings fairly for different variant types. We also applied a CADD cutoff of ≥15 to avoid individuals accruing high GenePy scores in genes of increasing length, where there was a higher chance of finding multiple ultrarare variants by pure chance that would score highly in GenePy. Although we are confident that using CADD ≥15 reduced a lot of noise and helped isolate pathogenic variants, we accept that this approach risks missing some pathogenic variants with lower CADD scores.

Fifty percent of individuals with a top 5 ranked GenePy score were unaffected. GenePy currently does not utilize phased data, meaning that some high scores may represent variants inherited in *cis*; indeed, we observed this in 71 cases (Table 1). However, we were conscious not to limit GenePy to nuclear families with parental data because this does not represent a real-world example and would disadvantage non-parent/child families for which phase cannot be determined. In the future, this could perhaps be mitigated with long read sequencing data.

Although we applied GenePy herein focusing on identification of potential missed recessive disease, there may also be opportunities to apply it in autosomal dominant diseases. When we scrutinized the variants of individuals with potential missed diagnoses, we identified 61 individuals that ranked in the top 5 GenePy scores for a given gene; yet, they only had one variant with a CADD score ≥15 in that gene. Most commonly, these individuals harbored predicted loss-of-function variants, which are upweighted in the GenePy statistic. Therefore, there may be utility of GenePy in haploinsufficient disease genes, but it is likely that a more stringent CADD cutoff, such as ≥20, or limiting the GenePy statistic to the highest scoring variant is necessary to apportion lower GenePy scores to individuals who would otherwise accrue high scores from multiple rare, but benign, variants with lower CADD scores.

GenePy also has potential to identify novel disease genes. If multiple top-ranking individuals across the same novel gene share similar clinical features, this may support the discovery of new disease genes. For novel haploinsufficient genes, unpublished data from our research group suggest that GenePy performs best when limited to high CADD scores, eg, CADD >20, whereas recessive genes may benefit from a more permissive CADD cutoff.

## Conclusion

The application of GenePy to ~78,000 individuals in the 100KGP has identified 122 putative missed biallelic diagnoses in known autosomal recessive disease genes that are being returned to participants through the Genomics England Diagnostic Discovery Pathway. Selecting the top 5 ranked individuals for 2864 autosomal recessive genes yielded review of only 1.2 additional variants per individual, rendering GenePy a useful tool to identify biallelic variants of interest without significantly burdening diagnostic laboratories with additional variants to assess. A dilemma for many diagnostic laboratories is how to limit number of variants requiring assessment without missing diagnoses. Although strategies to prioritize dominant diseases are well established, eg, de novo analysis or Exomiser,^16^ there are limited tools for prioritizing recessive conditions. We attest that GenePy is a useful panel-agnostic adjunct to exome and genome analysis pipelines to uplift diagnoses of recessive disease.

## Supplementary Material

Supplemental Results

Supplementary File 1

## Figures and Tables

**Table 1:** Flags applied to deprioritize variants

Variant Priority (No. of Variants)	At Least One Non-coding Variant	Common in Call-set	Does Not Segregate	In *cis*	No Second Hit
Top (122)	NA	NA	NA	NA	NA
Middle (262)	12	NA	NA	NA	NA
Low (72)	48	NA	NA	NA	NA
Exclude (229)	73	22	63	71	61

Variant pairs were deprioritized when at least 1 variant was non-coding on the MANE transcript, any variant was common in the 100,000 Genomes Project call-set (>5%), the variant(s) did not segregate between affected individuals from the same family, variants were in cis, or when only 1 heterozygous variant was identified.

## Data Availability

Access to the 100KGP data set analyzed in this study is only available as a registered GeCIP member in the Genomics England Research Environment, but restrictions apply to the availability of these data because of data protection and are not publicly available. Information regarding how to apply for data access is available at the following url: https://www.genomicsengland.co.uk/about-gecip/for-gecip-members/data-and-data-access/. Access to supplementary material and the full GenePy matrix is available within the Research environment at the following url:/re_gecip/shared_allGeCIPs/Ellie_Seaby/GenePy. All data shared in this manuscript were approved for export by Genomics England. The data sets and code supporting the current study are fully accessible within the Genomics England Research Environment.

## Abbreviations

100KGP: 100,000 Genomes Project
ACMG: American College of Human Genetics and Genomics
CADD: combined annotation dependent depletion
GEL: Genomics England GenCC, Gene Curation Coalition
GQ: genotype quality
HPO: human phenotype ontology
RE: research environment
OMIM: Online Inheritance in Man
MANE: matched annotation from NCBI and EMBL-EBI
